# Supplementary material for: Chromothripsis during telomere crisis is independent of NHEJ, and consistent with a replicative origin
Source: Genome Res. 2019 May;29(5):737–49. doi: 10.1101/gr.240705.118 (PMC6499312; doi:10.1101/gr.240705.118)
Supplement: Supplemental Material [file supp_gr.240705.118_Supplemental_file_1.zip › contigs/annotated_contigs/DB115/contig.2.DB115_length_262_mean_cov_19.0877862595.docx]

**DB115_length_262_mean_cov_19.0877862595**

```
CATATACACATATGTGTATATATGTGTACATATACATATACACATATGTGTATA|TATGTGTATATATACATATACACATATGT|GTAT
 >chrX:147662539-147662618 - E=3e-18 p=0e+00           >chrX:147662429-147662632 - E=5e
ATATGTGTGTATATATACATATGTACACATATGTGTATATATGTGTGTATATATACACATGTTTGTGTGTAGGAGGTGCATATATGTAT
-90
ATATTTGTGAGAGAGGGAAGAAGGAAGCAATGTGGTACAAATGTCAACATAGGAGGAATCTGGGTAATAGGGATATGAGAATTCTT
```
